# MiR-126-HMGB1-HIF-1 Axis Regulates Endothelial Cell Inflammation during Exposure to Hypoxia-Acidosis

**DOI:** 10.1155/2021/4933194

**Published:** 2021-12-21

**Authors:** Jinxue Liu, Eileen Wei, Jianqin Wei, Wei Zhou, Keith A. Webster, Bin Zhang, Dong Li, Gaoxing Zhang, Yidong Wei, Yusheng Long, Xiuyu Qi, Qianhuan Zhang, Dingli Xu

**Affiliations:** ^1^Department of Cardiology, Nanfang Hospital, Southern Medical University, Guangzhou 510515, China; ^2^Gulliver High School, Miami, FL 33156, USA; ^3^Department of Medicine Miller School of Medicine, University of Miami, Miami, FL 33136, USA; ^4^Department of Ophthalmology, Jiangmen Central Hospital, Affiliated Jiangmen Hospital of Sun Yat-Sen University, Jiangmen 529030, China; ^5^Integene International, LLC, Miami, FL 33137, USA; ^6^Cullen Eye Institute, Department of Ophthalmology, Baylor College of Medicine, Houston, TX 77030, USA; ^7^Everglades Biopharma, LLC, Houston, TX 77030, USA; ^8^Department of Cardiology, Jiangmen Central Hospital, Affiliated Jiangmen Hospital of Sun Yat-Sen University, Jiangmen 529030, China; ^9^Department of Intensive Care Unit and Clinical Experimental Center, Jiangmen Central Hospital, Affiliated Jiangmen Hospital of Sun Yat-Sen University, Jiangmen 529030, China; ^10^Department of Surgery, Youjiang Medical University for Nationalities, Chengxiang Rd, Baise, Guangxi 533000, China; ^11^Department of Cardiology, Guangdong Cardiovascular Institute, Guangzhou 510080, China; ^12^Department of Cardiology, Guangdong Cardiovascular Institute and Second School of Clinical Medicine, Southern Medical University, Guangzhou 510515, China; ^13^Department of Cardiology, Guangdong Cardiovascular Institute and Shantou University Medical College, Shantou 515041, China

## Abstract

Crosstalk between molecular regulators miR-126, hypoxia-inducible factor 1-alpha (HIF-1-*α*), and high-mobility group box-1 (HMGB1) contributes to the regulation of inflammation and angiogenesis in multiple physiological and pathophysiological settings. Here, we present evidence of an overriding role for miR-126 in the regulation of HMGB1 and its downstream proinflammatory effectors in endothelial cells subjected to hypoxia with concurrent acidosis (H/A). *Methods*. Primary mouse endothelial cells (PMEC) were exposed to hypoxia or H/A to simulate short or chronic low-flow ischemia, respectively. RT-qPCR quantified mRNA transcripts, and proteins were measured by western blot. ROS were quantified by fluorogenic ELISA and luciferase reporter assays employed to confirm an active miR-126 target in the HMGB1 3′UTR. *Results*. Enhanced expression of miR-126 in PMECs cultured under neutral hypoxia was suppressed under H/A, whereas the HMGB1 expression increased sequentially under both conditions. Enhanced expression of HMGB1 and downstream inflammation markers was blocked by the premiR-126 overexpression and optimized by antagomiR. Compared with neutral hypoxia, H/A suppressed the HIF-1*α* expression independently of miR-126. The results show that HMGB1 and downstream effectors are optimally induced by H/A relative to neutral hypoxia via crosstalk between hypoxia signaling, miR-126, and HIF-1*α*, whereas B-cell lymphoma 2(Bcl2), a HIF-1*α*, and miR-126 regulated gene expressed optimally under neutral hypoxia. *Conclusion*. Inflammatory responses of ECs to H/A are dynamically regulated by the combined actions of hypoxia, miR-126, and HIF-1*α* on the master regulator HMGB1. The findings may be relevant to vascular diseases including atherosclerotic occlusion and interiors of plaque where coexisting hypoxia and acidosis promote inflammation as a defining etiology.

## 1. Introduction

As integral vasoregulators, endothelial cells (ECs) serve as multifunctional biosensors that coordinate vascular responses to environmental stress of which hypoxia, oxidative stress, acidosis, and inflammation are especially prominent in myocardial disease and cancer [1–5]. By regulating EC survival, senescence, growth, invasion, glucose metabolism, and multiple molecular signaling pathways, hypoxia and HIF factor signaling are central to vascular EC responses to conditions of ischemia and downstream consequences of endothelial dysfunction, remodeling, and vascular disease [6–10]. Acidosis, an obligatory component of chronic ischemia caused by vessel occlusion, and present inside atherosclerotic plaque [11], is primarily driven by increased glycolysis and buildup of extracellular waste metabolites. Acidosis when combined with hypoxia additionally regulates and/or accentuates multiple aspects of the responses of ECs to ischemia, including survival, inflammation, and vessel tone and integrity via stress kinase signaling, calcium, and NO pathways [12–15]. Multiple microRNAs are known to modulate endothelial inflammatory responses [16] and established roles for miR-126 in regulating vascular integrity, angiogenesis, atherogenesis, and vessel functions that have been described [17–21].

Although miR-126 has been widely studied in the context of cellular hypoxia [22–27], its role in ECs subjected to chronic ischemic and/or acidotic conditions is relatively unexplored. HIF-1*α* has been shown to induce the miR-126 expression in a number of cell types including cultured human umbilical vein endothelial cells, and other studies have described positive feedback loop regulation between HIF-1*α* and miR-126 [26, 28, 29]. Consequently, HIF-1/miR-126 signaling is implicated in vasculogenesis and vascular disease, including proliferation, differentiation, migration, atherogenesis, senescence, and programmed cell death of vascular cells [30]. Inflammation is a fundamental cellular component of innate and adaptive immunity that, when deregulated is implicated in multiple cardiovascular pathologies, notably those that involve atherosclerosis, diabetes, obesity, hypertension, and responses to ischemia-reperfusion and myocardial infarction [31–33]. Acidosis occurs most frequently in association with sustained ischemia, inflammation, and metabolic disease where under the most severe conditions of ischemia, affected tissue pH can fall below 6.5 [12] and significantly impact basic physiological processes including immune responses, cell viability, angiogenesis, and localized inflammation [34–37].

The high-mobility group box 1 protein (HMGB1) is a secreted cytokine immunomodulator with central roles in autoimmune, infectious, and inflammatory pathologies especially related to cancer and cardiovascular disease. HMGB1 has been linked with angiogenesis, endothelial dysfunction, inflammation, and atherosclerosis through its regulation of toll-like receptor 4 and inflammatory cytokine secretions [38–43]. HMGB1 is expressed in myocardial cells where it selectively binds chromatin and activates innate immune and inflammatory-related genes [44]. Recently, microRNAs including miR-126 have been shown to confer important regulation of HMGB1 [45–50]. Here, we identify a pH component in the regulation of HMGB1 with contextual targeting by miR-126 that constitutes a critical component of signal transmission in the EC response to conditions of chronic simulated ischemia and associated inflammation.

## 2. Materials and Methods

### 2.1. Reagents

Primary mouse aortic endothelial cells (PMEC) were from Cell Biologics. Antibodies were obtained from the following vendors: p-Akt, Akt, Bcl2, TNF-*α*, and NAPDH oxidase, from Cell Signaling Technology, and HMGB1 and NADPH from Abcam; human premicroRNA expression constructs, Lenti-PremiR-126 and Anti-miR-126 from System Bioscience LLC; OxiSelect ROS assay kit from Cell Biolabs; Lipofectamine 2000 reagent from Thermo Fisher Scientific; and Luc-Pair miR luciferase assay kit from GeneCopoeia.

### 2.2. Endothelial Cell Culture and Treatment

PMECs, plated at 1 × 10^6^ cells per ml, were cultured in Dulbecco's Modified Eagle's Medium (DMEM) with 10% fetal bovine serum in a humidified atmosphere with 5% CO_2_ at 37°C. Our conditions for exposure to hypoxia (0.5% O_2_/5% CO_2_) are described in detail elsewhere [51–53]. Media was titrated with lactic acid to achieve a starting pH of 7.4 ± 0.05 for hypoxia alone and 6.7 ± 0.05 for hypoxia-acidosis (H/A), a moderately acidic pH for ischemic tissues within the range that can be caused by severely occluded myocardial tissue or within a tumor environment in vivo [54, 55]. Our H/A conditions are designed to mimic chronic low-flow ischemia caused by such severe occlusion as well as ECs within an atherosclerotic lesion where oxygen and ionic exchanges between vessels and the blood are restricted. Media for the H/A condition was replaced daily, and cultures were exposed to hypoxia for 24 h and H/A for 72 h to more closely mimic chronic ischemia. Previous studies by others and ourselves have documented that most cells including primary ECs respond rapidly to hypoxia with activation of HIF-1*α* within 8-12 h of exposure and minimal additional change of HIF-1 responses between 24 and 72 h [56–62]. Under these conditions, we found that media pH under either condition did not change significantly over 24 h. Extended times are also appropriate to mimic metabolic adaptations to simulated ischemia because of the vastly larger extracellular space of cultured ECs versus vascular ECs in vivo. In some incubations, cells were subjected to lentivirus infection using Lipofectamine 2000 before exposure to conditions.

### 2.3. Western Blot

Our procedures for western blots are described in detail elsewhere [51, 52]. Briefly, 30 *μ*g of total protein per lane in loading buffer was separated by 12% SDS-PAGE gel and proteins transferred onto membranes. After blocking, primary antibodies (1 : 500 dilution) were incubated overnight at 4°C, followed by room temperature exposure to secondary antibodies (1 : 4000 dilution). Reactive bands were revealed by chemiluminescence.

### 2.4. RNA Analysis

For RNA quantification, total RNA was isolated from cells using TRIzol Reagent and subjected to real-time PCR using TaqMan probes (Applied Biosystems) as described previously [63]. All values are expressed relative to a mean expression value for the 22,000+ transcripts on each microarray.

### 2.5. Measurement of ROS

ROS were measured using an OxiSelect ROS assay kit, exactly as described by the manufacturer and as previously reported [64].

### 2.6. Luciferase Reporter Assay

Luciferase assays were performed on cell extracts as previously described [65] using a Luc-Pair miR luciferase assay kit (GeneCoepia). Relative luciferase activities are expressed as luminescence units normalized to Renilla luciferase activity. Luminescence was quantitated using a multimode microplate reader (BMG Labtech).

### 2.7. Quantitative RT-PCR

The MiR-126 expression was quantified using a quantitative real-time reverse transcription-PCR assay from Ambion described previously [66]. Briefly, PCR reactions were carried out in triplicate in a 25 *μ*l volume using SYBR Green Assay Master Mix (Applied Biosystems) for 3 min at 95°C, followed by 40 cycles of 95°C for 15 s, 60°C for 30 s, and 72°C for 45 s in a Bio-Rad I Cycler (Bio-Rad Laboratories). Micro-RNA primers were used as follows: miR-126 forward, 5′-TATAAGATCTGAGGATAGGTGGGTTCCCGAGAACT-3′, reverse, 5′-ATATGAATTCTCTCAGGGCTATGCCGCCTAAGTAC-3′; HMGB1 forward, 5′-TATGGCAAAAGCGGACAAGG-3′, reverse, 5′-CTTCGCAACATCACCAATGGA-3′; GAPDH forward, 5′-ACAACTTTGGTATCGTGGAAGG-3′, reverse, 5′-GCCATCACGCCACAGTTTC-3′; U6 forward 5′-CTCGCTTCGGCAGCACA-3′, reverse 5′-AACGCTTCACGAATTTGCGT-3′. The relative gene expression was quantified using the 2^−*ΔΔ*Cq^ method [67]. Three independent experiments were routinely performed for each assay.

### 2.8. Statistics

All data are expressed as mean ± S.E.M. Statistical comparisons were performed using Graphpad Prism software (GraphPad Software Inc.), and Student's *t*-test was used to compare differences.

## 3. Results

**Figure 1:**
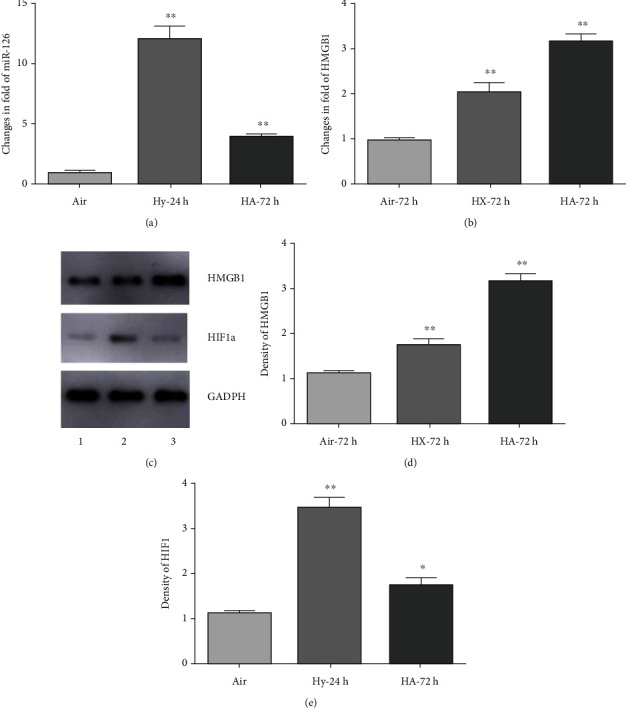
Quantification of responses of miR-126, HMGB1, and HIF-1*α* to hypoxia and H/A in PMECs. MiR-126 and HMGB1 RNAs were measured by RT-PCR (a, b) and Western blot (c)–(e). Results are expressed as mean ± SEM. ^∗∗^*p* < 0.01, ^∗^*p* < 0.05. Hy-24 h and HA-72 h: PMECs were exposed to hypoxia for 24 hours or H/A for 72 hours. Results are expressed as mean ± SEM. ^∗∗^*p* < 0.01; ^∗^*p* < 0.05; *n* = 4.

Suppression of hypoxia-induced miR-126 and HMGB1 by acidosis of PMECs: cultured PMECs were subjected to 24 h of hypoxia alone or 72 h hypoxia with concurrent acidosis and isolated RNAs and proteins quantified for expression of mir-126, HMGB1, and HIF-1*α*. As shown in Figure 1, hypoxia alone conferred increased expression of miR-126 and HMGB1-specific RNAs, respectively, by 12 ± 2 − fold and 2.1 ± 0.1 − fold (both *p* < 0.01 relative to aerobic controls) and proteins HMGB1 and HIF-1*α*, respectively, by 1.85 ± 0.1 − fold and 3.6 ± 0.2 − fold (both *p* < 0.01 relative to aerobic controls). When acidosis was present for 72 h with concurrent hypoxia, mir-126 levels were 4.1 ± 0.05 − fold relative to aerobic cells, a decline of 3-fold relative to hypoxia alone, whereas HMGB1 mRNA levels were further increased over pH neutral hypoxia to 3.2 ± 0.15 of aerobic cell, an increase of 50% over neutral hypoxia. At the protein level, HMGB1 protein increased in parallel with the mRNA also to 3.2 ± 0.1 − fold relative to aerobic cells, whereas HIF-1 protein under H/A was 1.8 ± 0.1 − fold of aerobic cells, a 50% decline relative to neutral hypoxia. As discussed in Methods, previous work by others and ourselves has shown that HIF-1*α* accumulates rapidly when primary ECs are exposed to hypoxia, maximally within 4-8 h with no significant change between 24 and 72 h in most cases [56, 57, 61]. The results indicate positive regulation of all 3 RNA/gene targets by hypoxia and quenching of miR-126 and HIF-1*α* by concurrent acidosis, but enhanced expression of HMGB1 by H/A.

### 3.1. Inflammatory Indicators, Increased under Hypoxia, Are Enhanced by HA

**Figure 2:**
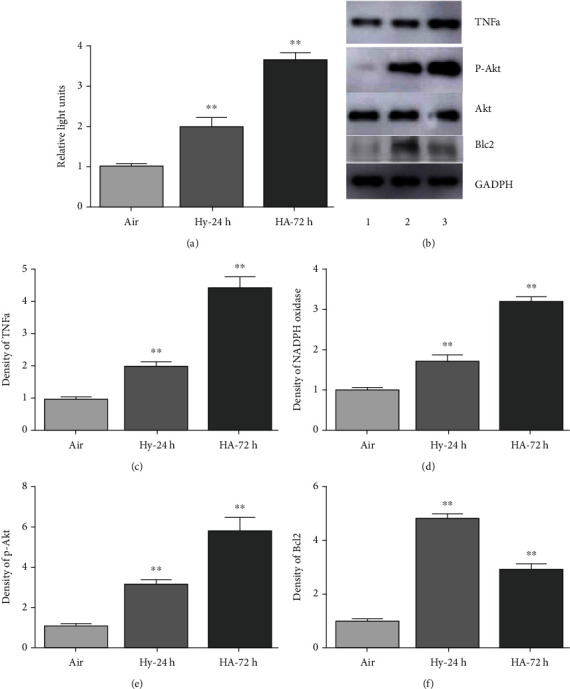
Responses of markers of inflammation, Akt, and Bcl2 during exposure of PMECs to 24 h hypoxia (Hy) and 72 h H/A. ECs were infected with premiR-126 or antagomiR-126 and cultured under the specified conditions. (a) ROS were quantified using an OxiSelect ROS assay kit. (b)–(f) Western blots quantified TNF*α*, p-Akt, and Bcl2. Results are expressed as mean ± SEM. ^∗∗^*p* < 0.01; ^∗^*p* < 0.05; *n* = 4.

HMGB1 is a secreted immune-inflammatory protein expressed in many cell types, that acts as a damage-associated molecular pattern (DAMP) factor [68] and can induce signaling pathways by binding to immune modulators such as advanced glycation end products (RAGE) and toll-like receptors (TLRs) [69, 70], thereby stimulating inflammatory cascade. To investigate functional consequences of HMGB1 induction by hypoxia in the presence and absence of acidosis, we assayed putative downstream inflammatory effectors of HMGB1 including ROS, NADPH, and TNF-*α*, as well as survival signaling pathway intermediates p-Akt and Bcl-2. As shown in Figure 2, ROS production, TNF-*α*, and NAPDH expression were significantly increased by both hypoxia alone and hypoxia-acidosis (*p* < 0.01), in a manner that paralleled closely the expression patters of HMGB1. P-Akt, a marker of survival kinases, was also significantly induced by both experimental manipulations while prosurvival, antiapoptosis marker Bcl2 was increased by both hypoxia and hypoxia-acidosis but more markedly by the former (all *p* < 0.01). The results are consistent with positive and regulation of HMGB1 and its downstream inflammatory effectors by hypoxia that is incrementally enhanced by concurrent acidosis.

### 3.2. Contextual Regulation of the HMGB1 Expression by miR-126 Modulators

**Figure 3:**
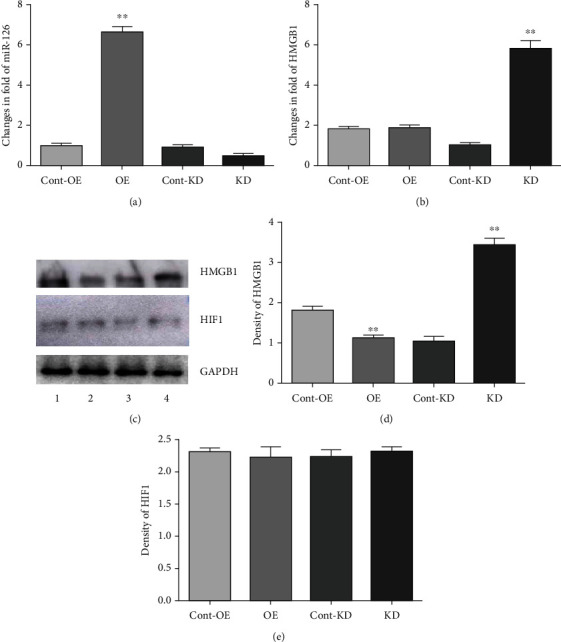
PremiR and antagomir regulation of miR-126 expression and downstream responses of HMGB1 and HIF-1*α* in PMECs during H/A culture. PMECs were infected with premiR-126 (OE) or anti-miR-126 (KD) and cultured under H/A. (a, b) miR-126 and HMGB1 RNAs were measured by RT-PCR. (c)–(e) western blots quantified and HIF-1*α*. Results are expressed as mean ± SEM. ^∗∗^*p* < 0.01. Cont-OE and Cont-KD refer to control (empty) vectors for the respective experimental premiR and antagomir overexpression groups. Results are expressed as mean ± SEM. ^∗∗^*p* < 0.01; ^∗^*p* < 0.01; *n* = 4.

It was reported that the HMGB1 gene contains a 3′UTR target for miR-126 and that elevated miR-126 downregulated HMGB1 and suppressed inflammatory responses of ECs during exposure to hyperglycemia [50]. Because the actions of miR-126 are context-dependent and can mediate positive or negative actions on gene expression depending on the prevailing environments [21], we asked whether miR-126 contributes to the incremental regulation of HMGB1 gene expression by hypoxia-acidosis. To do this, PMECs were transfected with optimal doses of miR-126 premiR or antagomir RNAs and the expression of HMGB1 and HIF-1*α* measured after exposure to H/A for 72 h as described in Methods. As shown in Figure 3, ECs pretransfected with the miR-126 mimic displayed robust expression of miR-126 relative to controls, whereas the expression in antagomir-transfected cells was reduced by about 50% of control nontransfected cells (Figure 3(a)). Despite the high expression of apparent miR-126 conferred by overexpression of the mimic, levels of HMGB1 mRNA remained unchanged relative to nontransfected control cells; perhaps, an indication that elevated basal miR-126 under hypoxia-acidosis alone is sufficient to convey significant degradation of HMGB1 mRNA. Conversely, the overexpression of the antagomir conferred almost 6-fold increased expression of HMGB1 mRNA; again, consistent with the possibility that endogenous miR-126 actively promotes degradation of HMGB1 mRNA expression under hypoxia-acidosis. Protein expression analyses supported such an interpretation that miR-126 regulates the HMGB1 gene expression during exposure to hypoxia-acidosis by promoting mRNA degradation and suppressing translation. As shown in Figures 3(c) and 3(d), the overexpression of the miR-126 mimic significantly blocked HMGB1 protein expression (*p* < 0.01), whereas the antagomir overexpression conferred >10-fold increased protein expression relative to control conditions. As a control, and to ensure that the results are not influenced by an indirect interference of miR-126 on HIF-1*α*, we demonstrate in Figure 3(e) that HIF-1*α* protein levels, increased under hypoxia-acidosis, were not affected by miR-126 mimic or antagomir overexpression in these ECs. The results demonstrate that decreasing miR-126 levels by transfection of an antagomiR prior to exposure to hypoxia-acidosis conferred markedly increased expression of HMGB1 mRNA and protein consistent with a classical miR-mediated targeting of the HMGB1 gene to induce mRNA degradation and translational repression [71]. This interpretation is also supported by the effects of the overexpression of the miR-126 mimic, although relatively minor compared with the antagomir. Both effects suggest significant regulation of the HMGB1 gene expression by endogenous miR-126 under hypoxia-acidosis.

### 3.3. Confirmation of a Hypoxia-Acidosis Regulable miR-126 Target Site on the HMGB1 Gene 3′UTR

**Figure 4:**
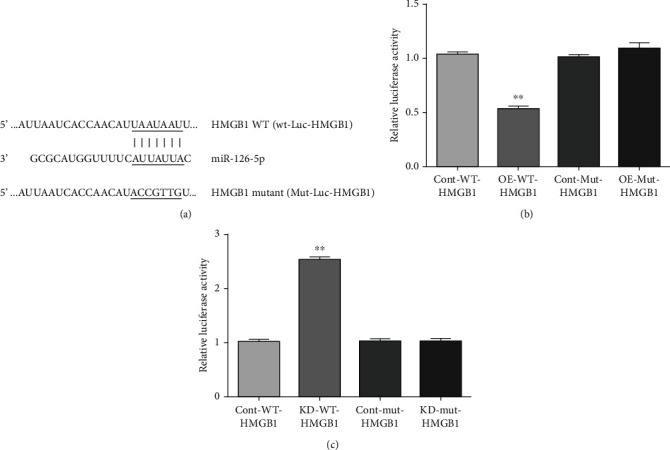
Context-dependent targeting of HMGB1 by miR-126. MirR-126 seed sequence and complementary binding site in the HMGB1 3′UTR are highlighted (a). PMECs were untreated or transfected with premiR-126 or antagomiR-126. Luciferase activities were quantified after subjection of cultures to H/A. Results are expressed as mean ± SEM. ^∗∗^*p* < 0.01; *n* = 4. Cont-WT-HMGB1: control (empty) vector for WT- HMGB1; OE-WT-HMGB1: overexpression of premiR-126 for WT HMGB1; Cont-Mut-HMGB1: control (empty) vector for mutant HMGB1; OE-Mut-HMGB1: overexpression of premiR-126 for mutant HMGB1; KD-WT-HMGB1: antagomiR-126 for WT-HMGB1; KD-Mut-HMGB1: antagomiR-126 for mutant HMGB1.

**Figure 5:**
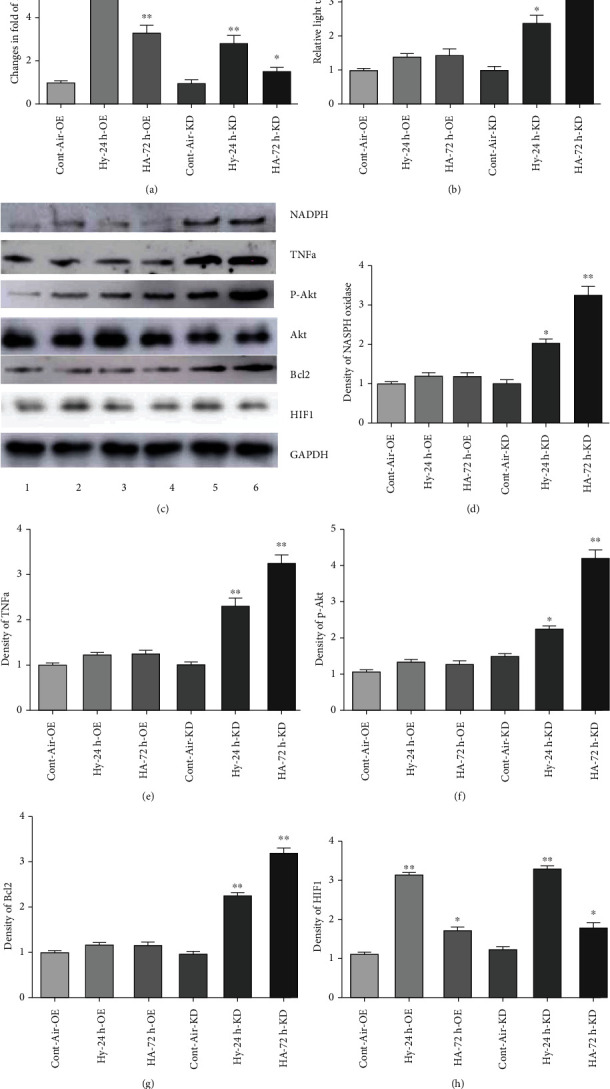
Attenuation of inflammatory markers in PMECs by miR-126 during H/A. ECs were infected with premiR-126 or antagomiR-126 and cultured under H/A. miR-126 RNA was measured using RT-PCR (a) ROS were measured using an OxiSelect ROS assay kit (b) Western blot analyses were performed to quantify TNF*α*, Akt, and Bcl2. (c)–(h) Results are expressed as mean ± SEM. ^∗∗^*p* < 0.01, ^∗^*p* < 0.01. Cont-Air-OE, Hy-24 h-OE, and HA-72 h-OE: ECs were transfected with empty vector or premiR-126 and treated aerobically. Hypoxia 24 h or H/A 72 h. Cont-Air-KD, Hy-24 h-KD, and HA-72 h-KD: ECs were transfected with control vector or antagomiR-126 and treated aerobically. Hypoxia 24 h and H/A 72 h.

To confirm that miR-126 can directly regulate the HMGB1 gene expression, we synthesized oligonucleotides containing putative wild type and mutant HMGB1-miR-126 binding sites and inserted them upstream of the Luciferase reporter gene as described in Methods (Figure 4(a)). Plasmids were transfected into PMECs with nontransfected cells as controls and subjected to conditions of hypoxia-acidosis (Figures 4(b) and 4(c)). We first confirmed that the overexpression of the premiR-126 conferred decreased expression of HMGB1, whereas knockdown by the antagomir conferred increased expression, as expected from results shown in Figures 1 and 3 (data not shown). Luciferase reporter gene assays revealed that luciferase activity was significantly decreased or enhanced, respectively, by premiR-126 or antagomiR-126, when compared with controls (*p* < 0.01). Importantly, the expression of an HMGB1 reporter plasmid that contained a mutated 3′UTR reporter gene was unaffected by either premiR-126 or antagomir (*p* > 0.5) (Figures 5(b) and 5(c)). These results confirm that miR-126 targets the HMGB1 gene expression through a 3′ UAAUAAUU target site and its regulation by H/A.

### 3.4. Predominant Role for miR-126 in the Regulation of Inflammation Markers by Hypoxia and H/A

To investigate possible individual roles of hypoxia, acidosis, HIF-1a, and the apparent overriding actions of miR-126 in regulating the HMGB1-responsive inflammatroy cascade, MPECs were pretransfected with miR-126 premiR or antagomiR, subjected to conditions of hypoxia alone or H/A and expression of inflammatory and cell survival markers measured and compared with aerobic controls as described in Methods and Figure 3. Relative intracellular levels of miR-126 after transfection of antagomiR or antagomiR and subjection to conditions are shown in Figure 5(a). Compared with antagomiR-overexpressing control aerobic incubations, exposure of transfected cells to hypoxia conferred increased miR-126 of 5.3 ± 0.3 − fold, that decreased to 3.4 ± 0.02 − fold under H/A. In antagomiR-transfected cells, miR-126 levels under hypoxic incubations were increased by 2.5 ± 0.2 − fold over aerobic controls and by 1.6 ± 0.1 − fold under H/A. The expression of inflammation markers, ROS, NADPH oxidase, TNF*α*, and survival kinase p-Akt displayed trends that are consistent with the results of Figure 3 and supports a major role for miR-126 in suppression of the EC inflammatory response via HMGB1 under conditions of hypoxia and especially H/A. The expressed levels of all proteins from cells incubated under hypoxia alone or H/A were the lowest when miR-126 was induced (Figures 5(b) and 5(d)–5(f), OE columns 2-3), and, conversely, the highest when miR-126 was reduced (Figures 5(b) and 5(d)–5(f), KD columns 5-6) consistent with an overriding role for miR-126 in the regulation and suppression by acidosis as a major component of inflammatory pathway regulation during H/A. Levels of the antiapoptosis survival protein Bcl2, induced under hypoxia, were further induced by H/A in the presence of miR-129 KD and low miR-129, consistent with the suppressive role for the miR-126 in Bcl2 gene expression [72, 73]. In agreement with the results shown in Figures 1 and 3, HIF-1*α* induction by hypoxia was reduced under the H/A condition and unaffected by OE or KD of miR-126 premiR (OE) or antagomiR (KD).

## 4. Discussion

**Figure 6:**
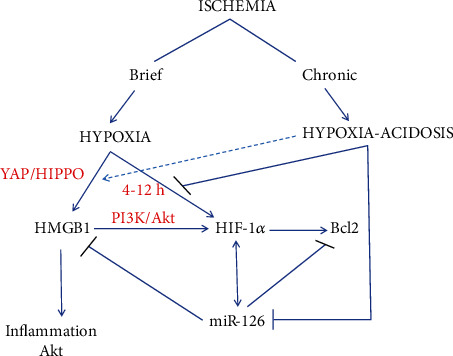
Schematic of crosstalk regulation of HMGB1, Akt, and Bcl2 expression and downstream inflammation mediators during exposure of PMECs to simulated ischemia. Acidosis is usually associated with severe, sustained ischemia when metabolic waste products including lactic acid accumulate within ischemic tissues. Hypoxia with or without acidosis rapidly activates HIF pathways, and miR-126 is induced in parallel with accumulated nuclear HIF-1*α* that increases the HMGB1 expression independently of HIF via YAP/HIPPO signaling, an effect that is retained under H/A (dashed line). By activating the PI3K/Akt pathway, HMGB1 also indirectly increases the HIF-1*α* expression in a hypoxia-HMGB1-HIF amplifying loop. HIF-1*α* directly and positively upregulates Bcl2, an effect that is countered via negative regulation by miR-126. During H/A, HIF-1a and miR-126 both decrease at least in part because acidosis blocks HIF-1*α*, the principal pathway for hypoxia-mediated transcriptional induction of miR-126. Reduced miR-126 during H/A relieves negative regulation on HMGB1 while the positive regulation by hypoxia is retained resulting in optimal activation of HMGB1 and its downstream inflammatory intermediates by H/A. In contrast because Bcl2 is positively regulated by HIF-1*α* and negatively regulated by miR-126, the optimal expression occurs under neutral hypoxia when HIF-1*α* is most active and predominates over miR-126.

We provide novel evidence for a dominant role of the miR-126 suppression by acidosis in the regulation of the HMGB1 gene expression and its downstream inflammation mediators in endothelial cells subjected to simulated chronic ischemia. The regulation is transmitted via a contextually responsive miR-126 target in the HMGB1 3′UTR. We also describe enhanced activation of the prosurvival, antiapoptosis protein Bcl2 by H/A via a slightly divergent signaling pathway that involves a quantitative antagonism between miR-126 and HIF-1*α*, whereas previous reports have documented negative regulation of both HMGB1 and Bcl2 by miR-126, and the present study is the first to describe acidosis as a driving force behind such regulation in the context of ischemia. Our findings that miR-126 levels increased 12-fold under pH-neutral hypoxia in parallel with a 3.5-fold increase of HIF-1*α* (Figures 1(a) and 1(e)) are consistent with previous reports that miR-126 is positively regulated by HIF-1*α* [26, 28, 29]. Previous work has also shown that HIF-1*α* can regulate micro-RNAs directly by binding to HRE-motifs in the 5′ upstream sequences of host genes [74], or indirectly by regulating the activities of associated signal intermediates, for example, c-Myc [75]. We found that the incremental increase of miR-126 supported by neutral hypoxia was reduced >70% under H/A, coincident with a similar >70% loss of the HIF1-*α* expression (Figures 1(a) and 1(e)), in parallel with a significant 50% augmentation of the HMGB1 expression under H/A relative to neutral hypoxia (*p* < 0.01). The results support an indirect role for HIF-1*α* in the regulation of HMBG1 via downregulation of miR-126. In contrast, the Bcl2 expression, optimally activated by neutral hypoxia, was reduced under H/A (Figures 1(a) and 2(f)), most likely due to the opposing effects of coincident downregulated HIF-1*α* and suppressed miR-126 under H/A (see illustration, Figure 6).

Results of premiR/antagomiR transfections confirmed the potent regulation of the HMGB1 gene expression by miR-126 during exposure to H/A (Figure 3). The HMBG1 expression was low in cells transfected with premiRs and maximally induced by antagomir (Figure 3(e) columns 2 and 4), whereas HIF-1*α* was unresponsive to premiR/anti-miR-modulated expression of miR-126 (Figure 3(e)). Cotransfection of PMECs with miR-126 premiR, antagomiR, and expression vectors directing expression of Luciferase by 5′ wild type or mutated miR-126 target sites confirmed an miR-126 target sequence in the HMGB1 3′UTR, as previously reported [50] and its responsiveness to H/A. Luciferase activity was differentially regulated by a factor > 5 − fold by WT premiR over antagomiR in transfected cells exposed to H/A (*p* < 0.01), and the regulation was eliminated by mutation of the site (Figure 4(b)). These results confirm the presence of a pH-regulable miR-126 target on the HMGB1 3′UTR.

The expression levels of HMGB1-regulated inflammation markers in transfected cells subjected to conditions of hypoxia and H/A followed patterns that are consistent with positive and negative regulation by hypoxia and miR-126, respectively (Figures 4(b)–4(e)). The HMGB1 gene is positively regulated by hypoxia through PI3K and YAP/Hippo pathways, that are independent of HIF-1*α*, and HMGB1 positively regulates the expression of HIF-1*α* [76–81]. Consistent with this, the inflammatory marker expression was only partially eliminated by miR-126 premiR transfection (OE) of cells under neutral hypoxia, compared with aerobic cells (Figures 4(b)–4(e), columns 1, 2, and 4), and the highest levels of inflammatory marker expression were seen in antagomiR expressing cells under H/A, a condition that drives maximal suppression of miR-126, sustained hypoxia, and reduced HIF-1*α*. It is noteworthy that Bcl2 expression, dually regulated in an antagonistic manner by HIF-1*α* and miR-126, was increased under H/A compared with neutral hypoxia only in antagomir-transfected cells (compare Figures 2(f) and 4(g)), despite lower HIF-1*α* (Figure 4(h)), suggesting an overriding role for miR-126 vs. HIF-1*α* in Bcl2 regulation under these conditions of simulated ischemia.

Taken together, the results support the scheme depicted in Figure 6. Neutral hypoxia increases miR-126 by HIF-1*α* dependent and independent pathways, and this induction is largely reversed by H/A. HMGB1, its downstream inflammatory markers and Akt, induced by neutral hypoxia, is super induced by HA due to H/A suppression of inhibitory miR-126 and sustained regulation by hypoxia via YAP/Hippo signaling. Repression of HIF-1*α* activity by H/A may be partially alleviated in this condition via positive feedback regulation by HMGB1, as well as positive regulation by miR-126 under some circumstances. Survival, antiapoptosis factor Bcl2 is directly regulated by HIF-1*α* and negatively regulated by miR-126; therefore, the relative levels of these regulators determine the Bcl2 expression. Consequently, neutral hypoxia conferred higher expression of Bcl2 than H/A, but H/A in the presence of miR-126 antagomiR conferred the greatest level of the Bcl2 expression.

The studies are relevant to inflammation involving the endothelium, especially during cardiovascular disease and rapidly growing tumors wherein microenvironments of hypoxia and acidosis are common [12–16, 82]. Previous work has also shown that proinflammatory factors are increased by an acidotic extracellular environment with or without hypoxia [83, 84]. Development of inflammation during sever chronic ischemia in both conditions is exacerbated by acidic pH as well as by the underlying hypoxia. Such changes interfere with a wide range of immunological functions conferring increased levels of inflammatory cytokines, interleukin IL-1*β*, IL-6, IL-8, IL-10, TNF-*α*, NADPH oxidase, and ROS [12, 82, 85].

In addition to targeting HMGB1, a key driver of inflammatory and immune responses in numerous disease settings [86–89], miR-126 can target multiple other disease-associated genes of relevance to this study, including angiogenesis-related vascular endothelial growth factor A, sprouty-related protein-1, phosphoinositidol-3 kinase regulatory subunit-2 1, and the adapter molecule crk [90–92]. Our discovery that acidosis exerts marked control over HMBG1 and Bcl2 expression as well as downstream inflammation responders and Akt, via miR-126 and HIF-1*α* (summarized in Figure 6), in the context of chronic ischemia is novel and represents important additions to our understanding of vascular inflammation and cell survival during ischemic disease, including atherosclerosis and vasculatures of solid tumors, wherein hypoxia and acidosis are integral disease components.

### 4.1. Study Limitations

Our results support an overriding role for miR-126 in optimally inducing expression of the HMGB1 gene and its downstream mediators of inflammation and oxidative stress during exposure to conditions of chronic simulated ischemia in cultured endothelial cells. The proposed mechanism involves acidosis-mediated suppression of HIF-1*α* activity and consequential blocking of hypoxia-induced transcription of the miR-126 host genes, thereby blocking HMGB1 suppression by mir-126. We acknowledge that the relation of HIF-1*α* with miR-126 is correlative, and we do not yet have a mechanism; however, the results are consistent with other reports that have described HIF-1*α* regulation of miR-126 in endothelial cells [26, 28, 29]. Also, we do not know the mechanism of acidosis regulation of HIF-1*α*; indeed, early literature has documented positive regulation of the HIF pathway by driving nuclear sequestration of the VLH factor [93]. Our unique conditions of chronic H/A may account for the differences. Finally, our use of a prolonged 72 h exposure time for H/A to more closely simulate chronic ischemia with acidosis introduces another variable. We contend that the comparison with 24 h neutral hypoxia is valid at least in part because previous reports by others and ourselves have shown that the HIF pathway of cultured endothelial cells responds rapidly to hypoxia, usually within 4-8 h, and is sustained without significant change during exposure times of 24 h through 72 h, and provided nutrients are replenished and media pH controlled, as is the case in our incubations [10, 56, 61, 62]. Except miRNA, other types of noncoding RNA may be associated with endothelial cell inflammation during exposure to H/A, including Piwi RNA [94] and circular RNA [95]. Further discussion is needed in future studies.

## Data Availability

The datasets generated during and/or analyzed during the current study are available from the corresponding author on reasonable request.

## References

[1] Wong B. W., Marsch E., Treps L., Baes M., Carmeliet P. (2017). Endothelial cell metabolism in health and disease: impact of hypoxia. *The EMBO Journal*.

[2] Eskin S. G., Ives C. L., McIntire L. V., Navarro L. T. (1984). Response of cultured endothelial cells to steady flow. *Microvascular Research*.

[3] Bouis D., Kusumanto Y., Meijer C., Mulder N., Hospers G. (2006). A review on pro- and anti-angiogenic factors as targets of clinical intervention. *Pharmacological Research*.

[4] Reriani M. K., Lerman L. O., Lerman A. (2010). Endothelial function as a functional expression of cardiovascular risk factors. *Biomarkers in Medicine*.

[5] Vestweber D. (2015). How leukocytes cross the vascular endothelium. *Nature Reviews. Immunology*.

[6] Janaszak-Jasiecka A., Siekierzycka A., Płoska A., Dobrucki I. T., Kalinowski L. (2021). Endothelial dysfunction driven by hypoxia-the influence of oxygen deficiency on NO bioavailability. *Biomolecules*.

[7] Ullah K., Wu R. (2021). Hypoxia-inducible factor regulates endothelial metabolism in cardiovascular disease. *Frontiers in Physiology*.

[8] Meyer M., Allenbach G., Nicod Lalonde M., Schaefer N., Prior J. O., Gnesin S. (2020). Increased ^18^F-FDG signal recovery from small physiological structures in digital PET/CT and application to the pituitary gland. *Scientific Reports*.

[9] Meyer L. (1976). Apartment clinics keep senior citizens in the community. *Hospitals*.

[10] Hu J., Discher D. J., Bishopric N. H., Webster K. A. (1998). Hypoxia regulates expression of the endothelin-1 gene through a proximal hypoxia-inducible factor-1 binding site on the antisense strand. *Biochemical and Biophysical Research Communications*.

[11] Ferns G., Heikal L. (2017). Hypoxia in Atherogenesis. *Angiology*.

[12] Crimi E., Taccone F. S., Infante T., Scolletta S., Crudele V., Napoli C. (2012). Effects of intracellular acidosis on endothelial function: An overview. *Journal of Critical Care*.

[13] Rohwer N., Cramer T. (2011). Hypoxia-mediated drug resistance: novel insights on the functional interaction of HIFs and cell death pathways. *Drug Resistance Updates*.

[14] Thompson J. W., Graham R. M., Webster K. A. (2012). DNase activation by hypoxia-acidosis parallels but is independent of programmed cell death. *Life Sciences*.

[15] Graham R. M., Frazier D. P., Thompson J. W. (2004). A unique pathway of cardiac myocyte death caused by hypoxia–acidosis. *The Journal of Experimental Biology*.

[16] Xiao L., Liu Y., Wang N. (2014). New paradigms in inflammatory signaling in vascular endothelial cells. *American Journal of Physiology. Heart and Circulatory Physiology*.

[17] Kuhnert F., Mancuso M. R., Hampton J. (2008). Attribution of vascular phenotypes of the murineEgfl7locus to the microRNAmiR-126. *Development*.

[18] Wang S., Aurora A. B., Johnson B. A. (2008). The endothelial-specific microRNA miR-126 governs vascular integrity and angiogenesis. *Developmental Cell*.

[19] Agudo J., Ruzo A., Tung N. (2014). The miR-126-VEGFR2 axis controls the innate response to pathogen-associated nucleic acids. *Nature Immunology*.

[20] Schober A., Nazari-Jahantigh M., Wei Y. (2014). MicroRNA-126-5p promotes endothelial proliferation and limits atherosclerosis by suppressing Dlk1. *Nature Medicine*.

[21] Nammian P., Razban V., Tabei S., Asadi-Yousefabad S. L. (2020). MicroRNA-126: dual role in angiogenesis dependent Diseases. *Current Pharmaceutical Design*.

[22] Alique M., Bodega G., Giannarelli C., Carracedo J., Ramírez R. (2019). MicroRNA-126 regulates hypoxia-inducible factor-1*α* which inhibited migration, proliferation, and angiogenesis in replicative endothelial senescence. *Scientific Reports*.

[23] Pan Q., Wang Y., Lan Q. (2019). Exosomes derived from mesenchymal stem cells ameliorate hypoxia/reoxygenation- injured ECs via transferring microRNA-126. *Stem Cells International*.

[24] Yang H. H., Chen Y., Gao C. Y., Cui Z. T., Yao J. M. (2017). Protective effects of MicroRNA-126 on human cardiac microvascular endothelial cells against hypoxia/reoxygenation-induced injury and inflammatory response by activating PI3K/Akt/eNOS signaling pathway. *Cellular Physiology and Biochemistry*.

[25] Ye P., Liu J., He F., Xu W., Yao K. (2014). Hypoxia-induced deregulation of miR-126 and its regulative effect on VEGF and MMP-9 expression. *International Journal of Medical Sciences*.

[26] Song W., Liang Q., Cai M., Tian Z. (2020). HIF-1*α*-induced up-regulation of microRNA-126 contributes to the effectiveness of exercise training on myocardial angiogenesis in myocardial infarction rats. *Journal of Cellular and Molecular Medicine*.

[27] Ren Y., Bao R., Guo Z., Kai J., Cai C. G., Li Z. (2021). miR-126-5p regulates H9c2 cell proliferation and apoptosis under hypoxic conditions by targeting IL-17A. *Experimental and Therapeutic Medicine*.

[28] Liu W., Li L., Rong Y. (2020). Hypoxic mesenchymal stem cell-derived exosomes promote bone fracture healing by the transfer of miR-126. *Acta Biomaterialia*.

[29] Ong S. G., Lee W. H., Huang M. (2014). Cross talk of combined gene and cell therapy in ischemic heart disease: role of exosomal microRNA transfer. *Circulation*.

[30] Chistiakov D. A., Orekhov A. N., Bobryshev Y. V. (2016). The role of miR-126 in embryonic angiogenesis, adult vascular homeostasis, and vascular repair and its alterations in atherosclerotic disease. *Journal of Molecular and Cellular Cardiology*.

[31] Elia E., Ministrini S., Carbone F., Montecucco F. (2021). Diabetic cardiomyopathy and inflammation: development of hostile microenvironment resulting in cardiac damage. *Minerva Cardioangiologica*.

[32] Wang M., Liu Y., Liang Y., Naruse K., Takahashi K. (2021). Systematic understanding of pathophysiological mechanisms of oxidative stress-related conditions-diabetes mellitus, cardiovascular diseases, and ischemia-reperfusion injury. *Frontiers in Cardiovascular Medicine*.

[33] Harrington D. L., Barten H. R., Audannio E. I. (2021). Genome sequences ofgordoniabacteriophages Jodelie19, BlingBling, and Burnsey. *Microbiology Resource Announcements*.

[34] Dong L., Krewson E. A., Yang L. V. (2017). Acidosis activates endoplasmic reticulum stress pathways through GPR4 in human vascular endothelial cells. *International Journal of Molecular Sciences*.

[35] Chen A., Dong L., Leffler N. R., Asch A. S., Witte O. N., Yang L. V. (2011). Activation of GPR4 by acidosis increases endothelial cell adhesion through the cAMP/Epac pathway. *PLoS One*.

[36] Dong L., Li Z., Leffler N. R., Asch A. S., Chi J. T., Yang L. V. (2013). Acidosis activation of the proton-sensing GPR4 receptor stimulates vascular endothelial cell inflammatory responses revealed by transcriptome analysis. *PLoS One*.

[37] Siesjö B. K., Katsura K. I., Kristián T., Li P. A., Siesjö P. (1996). Molecular mechanisms of acidosis-mediated damage. *Acta Neurochirurgica. Supplement*.

[38] Yang J., Huang C., Yang J., Jiang H., Ding J. (2010). Statins attenuate high mobility group box-1 protein induced vascular endothelial activation : a key role for TLR4/NF-*κ*B signaling pathway. *Molecular and Cellular Biochemistry*.

[39] Lin Q., Yang X. P., Fang D. (2011). High-mobility group box-1 mediates toll-like receptor 4-dependent angiogenesis. *Arteriosclerosis, Thrombosis, and Vascular Biology*.

[40] Bauer E. M., Shapiro R., Billiar T. R., Bauer P. M. (2013). High mobility group box 1 inhibits human pulmonary artery endothelial cell migration via a toll-like receptor 4- and interferon response factor 3-dependent mechanism(s). *The Journal of Biological Chemistry*.

[41] Yang S., Xu L., Yang T., Wang F. (2014). High-mobility group box-1 and its role in angiogenesis. *Journal of Leukocyte Biology*.

[42] Mudaliar H., Pollock C., Ma J., Wu H., Chadban S., Panchapakesan U. (2014). The role of TLR2 and 4-mediated inflammatory pathways in endothelial cells exposed to high glucose. *PLoS One*.

[43] Lan J., Luo H., Wu R. (2020). Internalization of HMGB1 (high mobility group box 1) promotes angiogenesis in endothelial cells. *Arteriosclerosis, Thrombosis, and Vascular Biology*.

[44] Wahid A., Chen W., Wang X., Tang X. (2021). High-mobility group box 1 serves as an inflammation driver of cardiovascular disease. *Biomedicine & Pharmacotherapy*.

[45] Liu L., Ren W., Chen K. (2017). MiR-34a promotes apoptosis and inhibits autophagy by targeting HMGB1 in acute myeloid leukemia cells. *Cellular Physiology and Biochemistry*.

[46] Coleman L. J., Zou J., Crews F. T. (2017). Microglial-derived miRNA let-7 and HMGB1 contribute to ethanol-induced neurotoxicity via TLR7. *Journal of Neuroinflammation*.

[47] Wang Y., Shen S., Li Z., Li W., Weng X. (2020). MIR-140-5p affects chondrocyte proliferation, apoptosis, and inflammation by targeting HMGB1 in osteoarthritis. *Inflammation Research*.

[48] Ai H., Zhou W., Wang Z., Qiong G., Chen Z., Deng S. (2018). microRNAs-107 inhibited autophagy, proliferation, and migration of breast cancer cells by targeting HMGB1. *Journal of Cellular Biochemistry*.

[49] Zhang W., Wang Y., Kong Y. (2019). Exosomes derived from mesenchymal stem cells modulate miR-126 to ameliorate hyperglycemia-induced retinal inflammation via targeting HMGB1. *Investigative Ophthalmology & Visual Science*.

[50] Tang S. T., Wang F., Shao M., Wang Y., Zhu H. Q. (2017). MicroRNA-126 suppresses inflammation in endothelial cells under hyperglycemic condition by targeting HMGB1. *Vascular Pharmacology*.

[51] Webster K. A., Discher D. J., Kaiser S., Hernandez O., Sato B., Bishopric N. H. (1999). Hypoxia-activated apoptosis of cardiac myocytes requires reoxygenation or a pH shift and is independent of p53. *The Journal of Clinical Investigation*.

[52] Kubasiak L. A., Hernandez O. M., Bishopric N. H., Webster K. A. (2002). Hypoxia and acidosis activate cardiac myocyte death through the Bcl-2 family protein BNIP3. *Proceedings of the National Academy of Sciences of the United States of America*.

[53] Webster K. A., Discher D. J., Hernandez O. M., Yamashita K., Bishopric N. H. (2000). A glycolytic pathway to apoptosis of hypoxic cardiac myocytes. Molecular pathways of increased acid production. *Advances in Experimental Medicine and Biology*.

[54] Swenson E. R. (2016). Hypoxia and its Acid-Base consequences: from mountains to malignancy. *Advances in Experimental Medicine and Biology*.

[55] Vaupel P., Multhoff G. (2018). Hypoxia-/HIF-1*α*-driven factors of the tumor microenvironment impeding antitumor immune responses and promoting malignant progression. *Advances in Experimental Medicine and Biology*.

[56] Bartoszewski R., Moszyńska A., Serocki M. (2019). Primary endothelial cell-specific regulation of hypoxia-inducible factor (HIF)-1 and HIF-2 and their target gene expression profiles during hypoxia. *The FASEB Journal*.

[57] Jin M. L., Zou Z. H., Tao T. (2020). Effect of the recombinant adenovirus-mediated HIF-1 alpha on the expression of VEGF in the hypoxic brain microvascular endothelial cells of Rats. *Neuropsychiatric Disease and Treatment*.

[58] Patel N., Gonsalves C. S., Malik P., Kalra V. K. (2008). Placenta growth factor augments endothelin-1 and endothelin-B receptor expression via hypoxia-inducible factor-1*α*. *Blood*.

[59] Kang B. Y., Kleinhenz J. M., Murphy T. C., Hart C. M. (2011). The PPAR*γ* ligand rosiglitazone attenuates hypoxia-induced endothelin signaling in vitro and in vivo. *American Journal of Physiology. Lung Cellular and Molecular Physiology*.

[60] Earley S., Nelin L. D., Chicoine L. G., Walker B. R. (2002). Hypoxia-induced pulmonary endothelin-1 expression is unaltered by nitric oxide. *Journal of Applied Physiology*.

[61] Luo J., Martinez J., Yin X., Sanchez A., Tripathy D., Grammas P. (2012). Hypoxia induces angiogenic factors in brain microvascular endothelial cells. *Microvascular Research*.

[62] Yamashita K., Discher D. J., Hu J., Bishopric N. H., Webster K. A. (2001). Molecular regulation of the endothelin-1 gene by hypoxia:. *The Journal of Biological Chemistry*.

[63] Wilson A., Shehadeh L. A., Yu H., Webster K. A. (2010). Age-related molecular genetic changes of murine bone marrow mesenchymal stem cells. *BMC Genomics*.

[64] Zhang B., Zhang G., Wei T. (2019). MicroRNA-25 protects smooth muscle cells against corticosterone-induced apoptosis. *Oxidative Medicine and Cellular Longevity*.

[65] Sharma S., Liu J., Wei J., Yuan H., Zhang T., Bishopric N. H. (2012). Repression of miR-142 by p300 and MAPK is required for survival signalling via gp130 during adaptive hypertrophy. *EMBO Molecular Medicine*.

[66] Shi H., Chen L., Wang H. (2013). Synergistic induction of miR-126 by hypoxia and HDAC inhibitors in cardiac myocytes. *Biochemical and Biophysical Research Communications*.

[67] Fang S., Ma X., Guo S., Lu J. (2017). MicroRNA-126 inhibits cell viability and invasion in a diabetic retinopathy model via targeting IRS-1. *Oncology Letters*.

[68] Bianchi M. E., Manfredi A. A. (2007). High-mobility group box 1 (HMGB1) protein at the crossroads between innate and adaptive immunity. *Immunological Reviews*.

[69] Harris H. E., Andersson U., Pisetsky D. S. (2012). HMGB1: a multifunctional alarmin driving autoimmune and inflammatory disease. *Nature Reviews Rheumatology*.

[70] Andersson U., Harris H. E. (2010). The role of HMGB1 in the pathogenesis of rheumatic disease. *Biochimica et Biophysica Acta*.

[71] Bartel D. P. (2018). Metazoan MicroRNAs. *Cell*.

[72] Gong C., Fang J., Li G., Liu H. H., Liu Z. S. (2017). Effects of microRNA-126 on cell proliferation, apoptosis and tumor angiogenesis via the down-regulating ERK signaling pathway by targeting EGFL7 in hepatocellular carcinoma. *Oncotarget*.

[73] Yu C. D., Miao W. H., Zhang Y. Y., Zou M. J., Yan X. F. (2018). Inhibition of miR-126 protects chondrocytes from IL-1*β* induced inflammation via upregulation of Bcl-2. *Bone and Joint Research*.

[74] Madej A., Popłoński J., Huszcza E. (2014). Improved oxidation of naringenin to carthamidin and isocarthamidin by Rhodotorula marina. *Applied Biochemistry and Biotechnology*.

[75] He M., Wang Q. Y., Yin Q. Q. (2013). HIF-1 *α* downregulates miR-17/20a directly targeting p21 and STAT3: a role in myeloid leukemic cell differentiation. *Cell Death and Differentiation*.

[76] Zhao M., Zhang Y., Jiang Y. (2021). YAP promotes autophagy and progression of gliomas via upregulating HMGB1. *Journal of Experimental & Clinical Cancer Research*.

[77] He H., Wang X., Chen J., Sun L., Sun H., Xie K. (2019). High-mobility group box 1 (HMGB1) promotes angiogenesis and tumor migration by regulating hypoxia-inducible factor 1 (HIF-1*α*) expression via the phosphatidylinositol 3-kinase (PI3K)/AKT signaling pathway in breast cancer cells. *Medical Science Monitor*.

[78] Xu Y. F., Liu Z. L., Pan C. (2019). HMGB1 correlates with angiogenesis and poor prognosis of perihilar cholangiocarcinoma via elevating VEGFR2 of vessel endothelium. *Oncogene*.

[79] Wang Z., Saadé N. K., Ariya P. A. (2021). Advances in ultra-trace analytical capability for micro/nanoplastics and water-soluble polymers in the environment: fresh falling urban snow. *Environmental Pollution*.

[80] Park S. Y., Lee S. W., Kim H. Y., Lee W. S., Hong K. W., Kim C. D. (2015). HMGB1 induces angiogenesis in rheumatoid arthritis via HIF-1*α* activation. *European Journal of Immunology*.

[81] Yao H. C., Zhou M., Zhou Y. H. (2016). Intravenous high mobility group box 1 upregulates the expression of HIF-1*α* in the myocardium via a protein kinase B-dependent pathway in rats following acute myocardial ischemia. *Molecular Medicine Reports*.

[82] Casimir G. J., Lefèvre N., Corazza F., Duchateau J., Chamekh M. (2018). The Acid-Base balance and gender in inflammation: a mini-review. *Frontiers in Immunology*.

[83] Iijima J., Konno K., Itano N. (2011). Inflammatory alterations of the extracellular matrix in the tumor microenvironment. *Cancers (Basel)*.

[84] Landskron G., de la Fuente M., Thuwajit P., Thuwajit C., Hermoso M. A. (2014). Chronic inflammation and cytokines in the tumor microenvironment. *Journal of Immunology Research*.

[85] Zampieri F. G., Kellum J. A., Park M. (2014). Relationship between acid-base status and inflammation in the critically ill. *Critical Care*.

[86] Deng C., Zhao L., Yang Z. (2021). Targeting HMGB1 for the treatment of sepsis and sepsis-induced organ injury. *Acta Pharmacologica Sinica*.

[87] Qu L., Chen C., Chen Y. (2019). High-mobility group box 1 (HMGB1) and autophagy in acute lung injury (ALI): a review. *Medical Science Monitor*.

[88] Liu Y., Zhuang G. B., Zhou X. Z. (2018). HMBG1 as a driver of inflammatory and immune processes in the pathogenesis of ocular diseases. *Journal of Ophthalmology*.

[89] Pilzweger C., Holdenrieder S. (2015). Circulating HMGB1 and RAGE as clinical biomarkers in malignant and autoimmune diseases. *Diagnostics (Basel)*.

[90] Fernández-Hernando C., Suárez Y. (2018). MicroRNAs in endothelial cell homeostasis and vascular disease. *Current Opinion in Hematology*.

[91] Fish J. E., Santoro M. M., Morton S. U. (2008). miR-126 regulates angiogenic signaling and vascular integrity. *Developmental Cell*.

[92] Theofilis P., Sagris M., Oikonomou E. (2021). Inflammatory mechanisms contributing to endothelial dysfunction. *Biomedicine*.

[93] Mekhail K., Gunaratnam L., Bonicalzi M. E., Lee S. (2004). HIF activation by pH-dependent nucleolar sequestration of VHL. *Nature Cell Biology*.

[94] Zheng S., Zheng H., Huang A. (2020). Piwi-interacting RNAs play a role in vitamin C-mediated effects on endothelial aging. *International Journal of Medical Sciences*.

[95] Huang A., Zheng H., Wu Z., Chen M., Huang Y. (2020). Circular RNA-protein interactions: functions, mechanisms, and identification. *Theranostics*.

